# Ulcerative Lesions in the Intertriginous Areas of an Adolescent Female Patient: A Case of Inverse Erosive Lichen Planus

**DOI:** 10.7759/cureus.76609

**Published:** 2024-12-30

**Authors:** Shivani Vasisht, Sushantika Sushantika, Poonam Saini, Prashant Durgapal, Soumya Nanda

**Affiliations:** 1 Dermatology, All India Institute of Medical Sciences, Rishikesh, Rishikesh, IND; 2 Pathology, All India Institute of Medical Sciences, Rishikesh, Rishikesh, IND

**Keywords:** dermoscopy, erosive, flexural, lichen planus, wickham striae

## Abstract

Lichen planus is a common mucocutaneous disorder that can affect various parts of the body, with its erosive variant typically involving the oral mucosa. This variant rarely affects the skin, and even less commonly, the flexural regions. Here, we present a case of a 14-year-old girl who presented with ulcerated, itchy lesions in the intertriginous areas for one year. Dermoscopic examination showed white, lacy reticular streaks suggestive of Wickham striae. Furthermore, on histopathology, the presence of interface dermatitis confirmed the diagnosis of inverse erosive lichen planus. This case underscores the role of dermoscopy in diagnosing rare erosive dermatoses in flexural regions, especially in pediatric patients. Prompt diagnosis, supported by histopathological and dermoscopic findings, facilitates early initiation of treatment. Our patient was treated with oral corticosteroids, topical clobetasol, and antihistamines, leading to complete resolution of the lesions.

## Introduction

Lichen planus is a chronic, immune-mediated disorder that affects the skin, mucosa, hair, and nails. It is characterized by pruritic, violaceous, polygonal papules and plaques, often accompanied by fine white lines known as Wickham striae. While it is relatively well-documented in adults, its occurrence in pediatric populations is uncommon [1]. Its pathogenesis remains incompletely understood, though it is believed to result from a complex interplay between genetic predisposition, environmental triggers, and immune system dysregulation. Cytotoxic T-cell-mediated damage to basal keratinocytes is considered a hallmark of the disease. This immune response may be triggered by factors such as viral infections, medications, or other environmental agents [2].

Lichen planus exhibits significant clinical heterogeneity, with various subtypes affecting different anatomical sites. Erosive lichen planus is a rare and challenging clinical entity. It most frequently affects mucosal surfaces, including the oral and genital mucosa, but erosive involvement of the skin is much less common. When it does occur, it typically affects acral sites such as the palms and soles [3]. Involvement of flexural areas is exceedingly rare, with only three adult cases reported in the literature to date [4-6]. These cases are often misdiagnosed as infectious or inflammatory dermatoses, such as scrofuloderma or condyloma lata, due to overlapping clinical features. Accurate diagnosis requires clinical correlation with dermoscopic and histopathological findings. Here, we report a novel case of inverse erosive lichen planus in an adolescent female patient, highlighting its atypical presentation, diagnostic challenges, and successful management. This case aims to broaden the understanding of this rare condition, emphasize the importance of considering inverse erosive lichen planus in the differential diagnosis of flexural dermatoses, and provide insights into potential therapeutic strategies for clinicians managing similar cases.

## Case presentation

A 14-year-old female patient presented with raw, ulcerated, itchy lesions in the groin, axilla, and perianal region, along with violaceous to brown itchy lesions on the nape of the neck, present for one year. These lesions appeared insidiously as dark-colored, raised areas and gradually increased in size and number. They were associated with significant pruritus, severe enough to disrupt sleep. Two months after onset, ulceration developed over the groin, perianal area, and axillary lesions, accompanied by a dirty yellow, non-foul-smelling purulent discharge from the inguinal fold lesion. There was no associated pain or bleeding. There was no history of fever, cough, weight loss, or other systemic symptoms. The patient was sexually inactive, with no significant past medical or surgical history.

**Figure 1:**
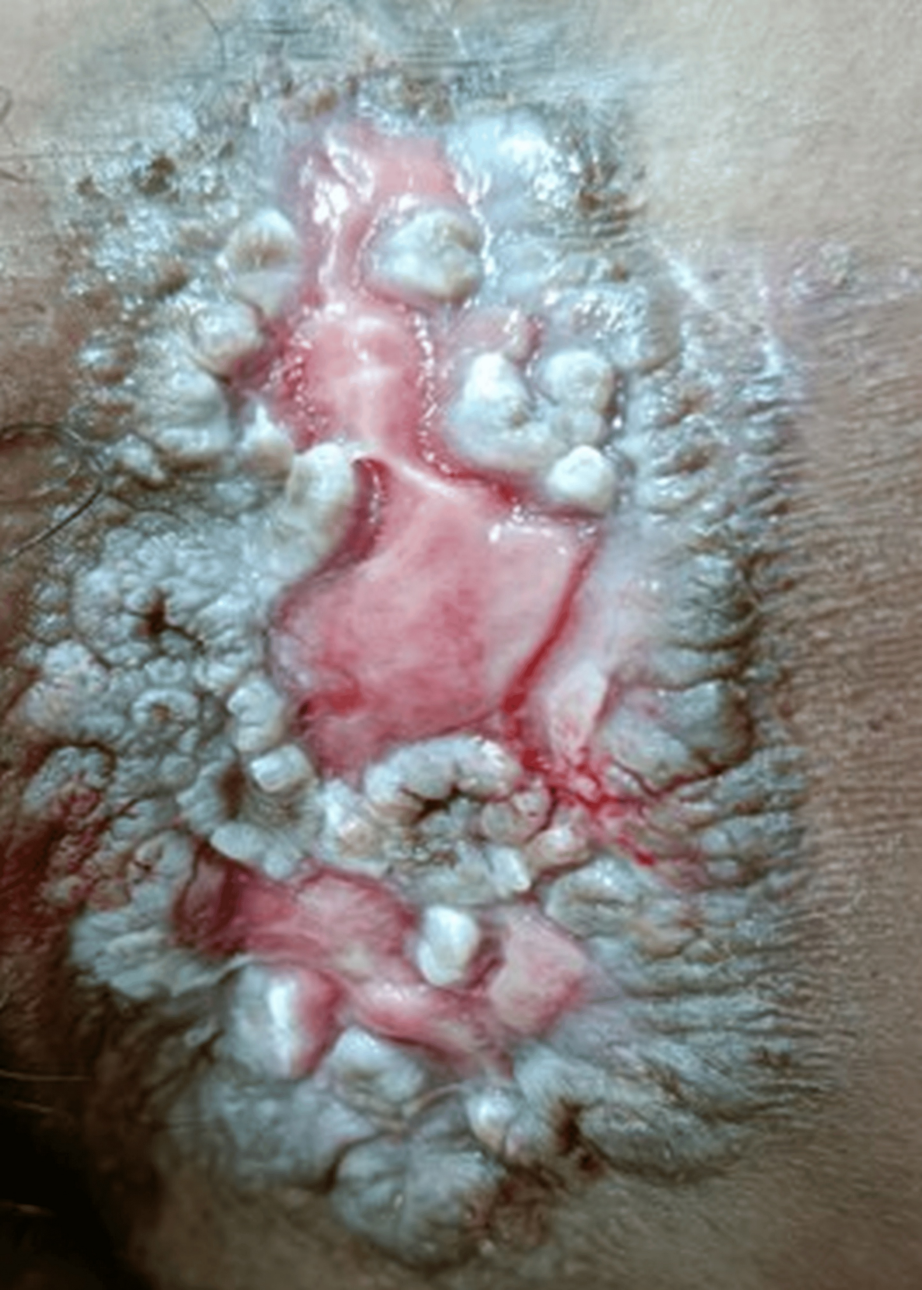
Well-defined ulcerated plaque with verrucous borders in the left inguinal fold.

**Figure 2:**
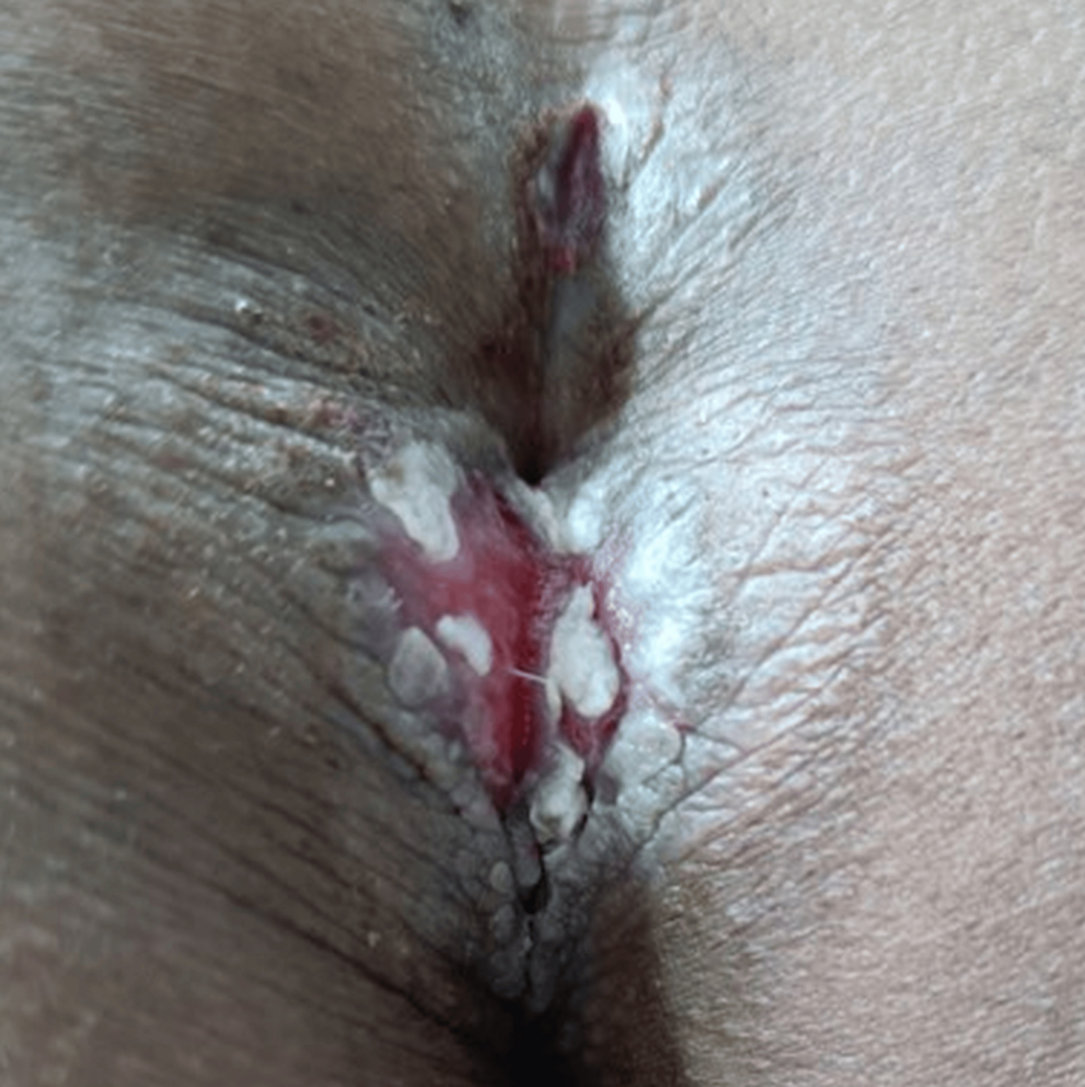
Hyperpigmented ulcerated plaque with verrucous borders in the perianal region.

**Figure 3:**
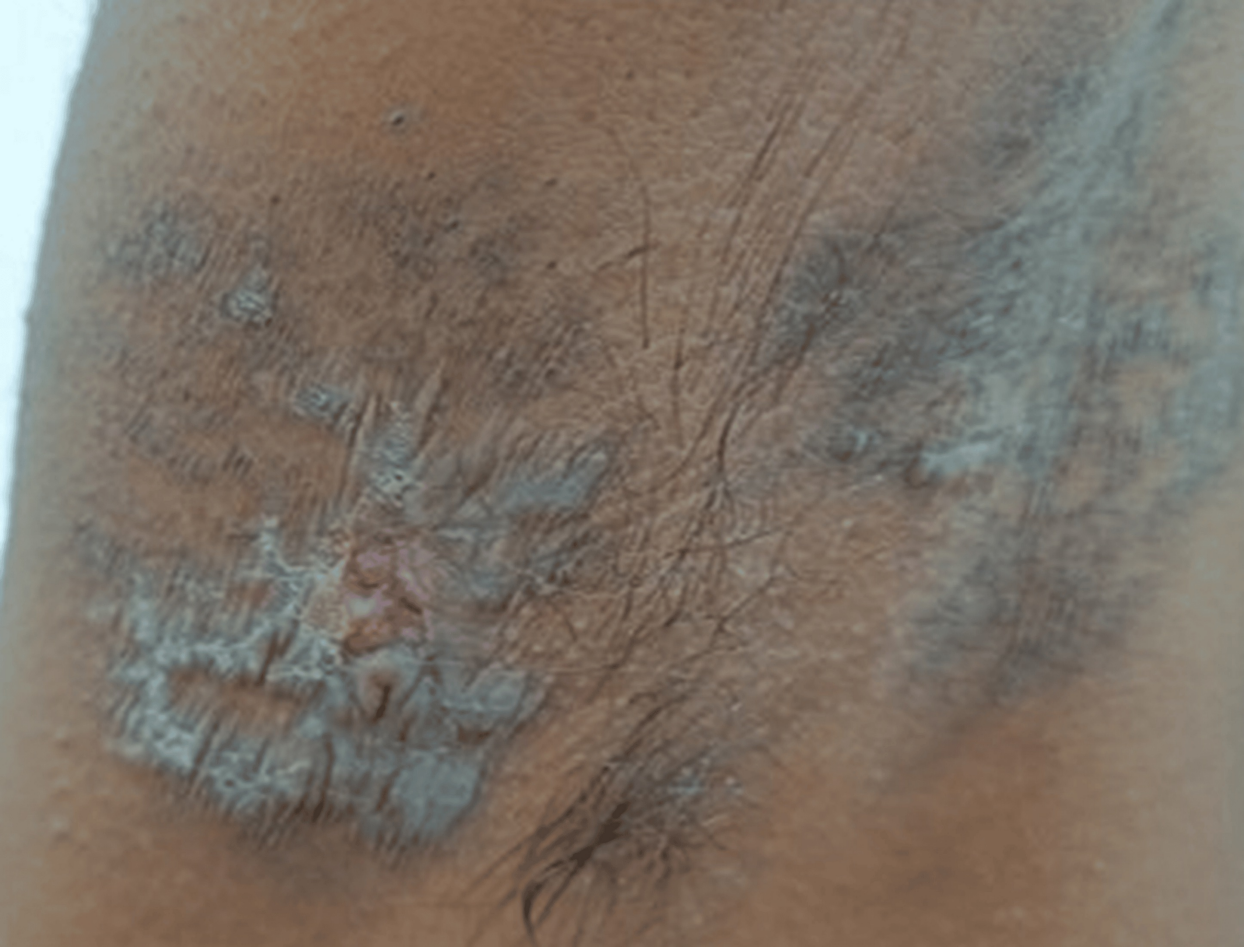
Violaceous to brown ulcerated plaques in the axilla. Lesions showing white, lacy reticular Wickham striae (lower left).

On clinical examination, there were multiple well-defined, non-tender, non-indurated plaques with irregular, hyperpigmented, verrucous borders and central ulceration, with a moist erythematous base. These were located on the left inguinal fold (Figure 1), gluteal cleft sparing the anal mucosa (Figure 2), and bilaterally in the axillae (Figure 3). 

**Figure 4:**
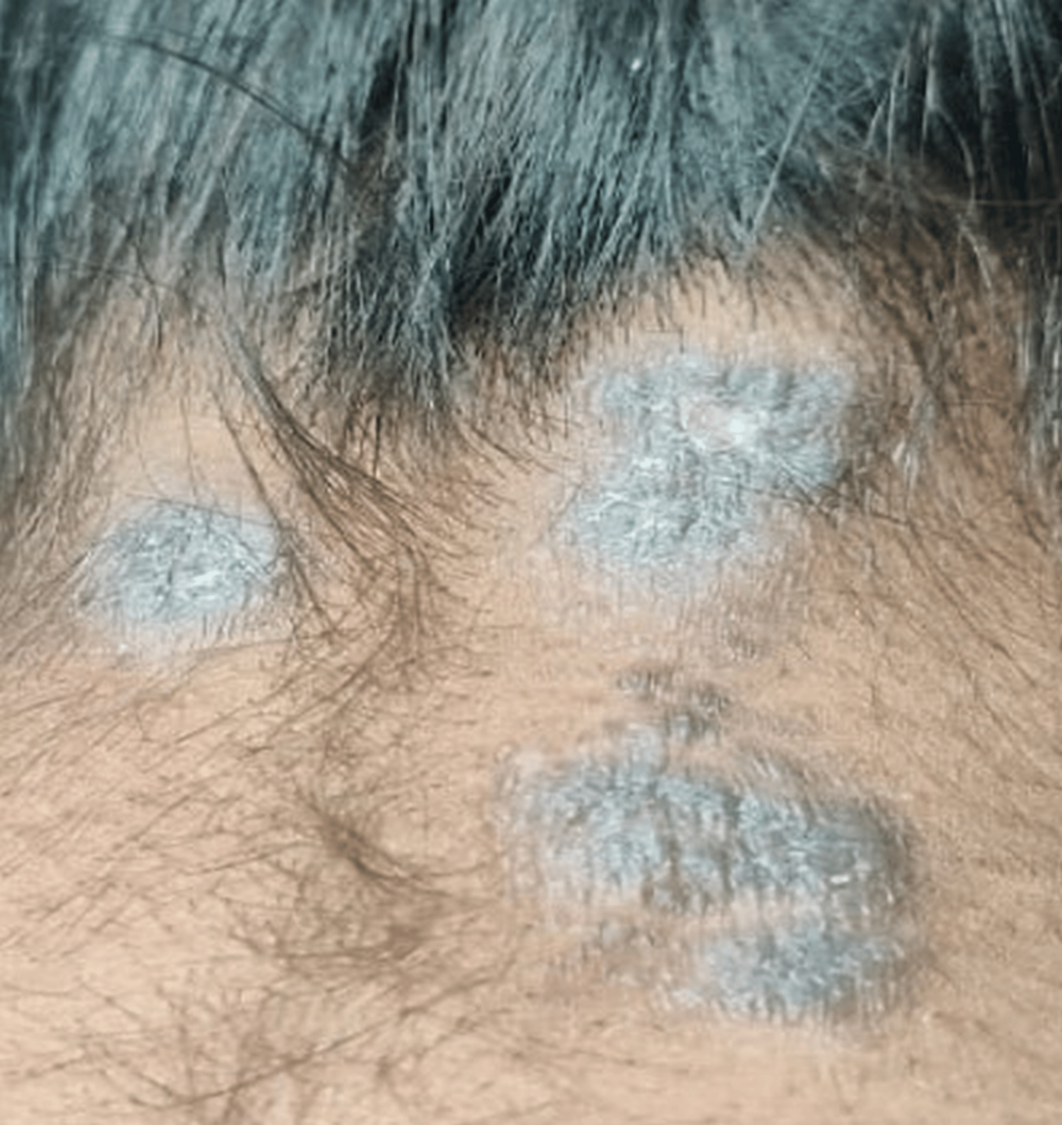
Multiple well-defined flat-topped hyperpigmented plaques with violaceous hue on the nape of the neck. Lesions showing white, lacy reticular Wickham striae.

Additionally, there were multiple well-defined, flat-topped, hyperpigmented plaques with a violaceous hue on the nape of the neck (Figure 4).

**Figure 5:**
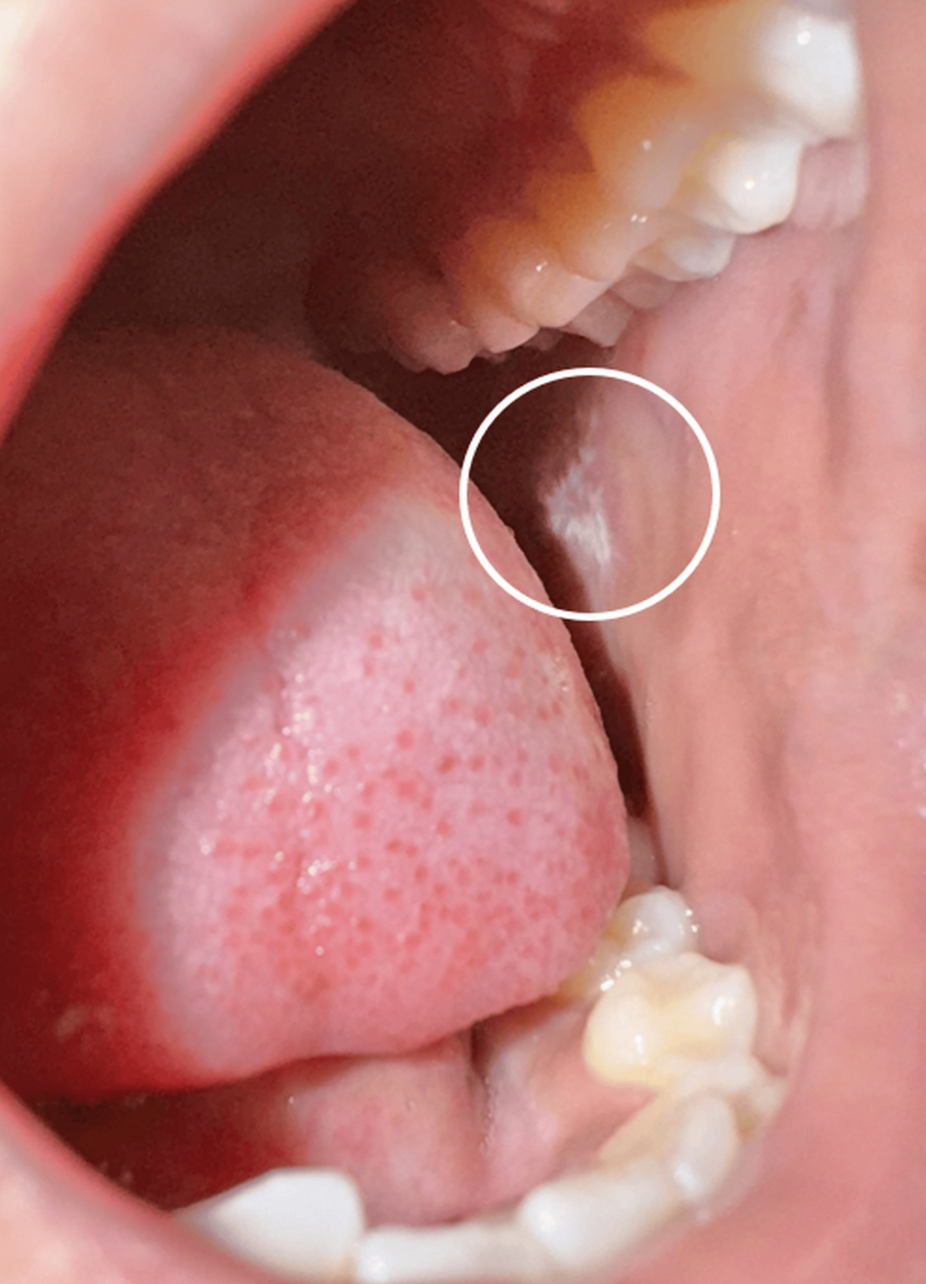
Oral mucosa showing white lacy reticulate striae over a violaceous base on the buccal mucosa.

Oral examination revealed white lacy reticulate striae over a violaceous base on the buccal mucosa (Figure 5). Rest of the mucosa were within normal limits.

**Figure 6:**
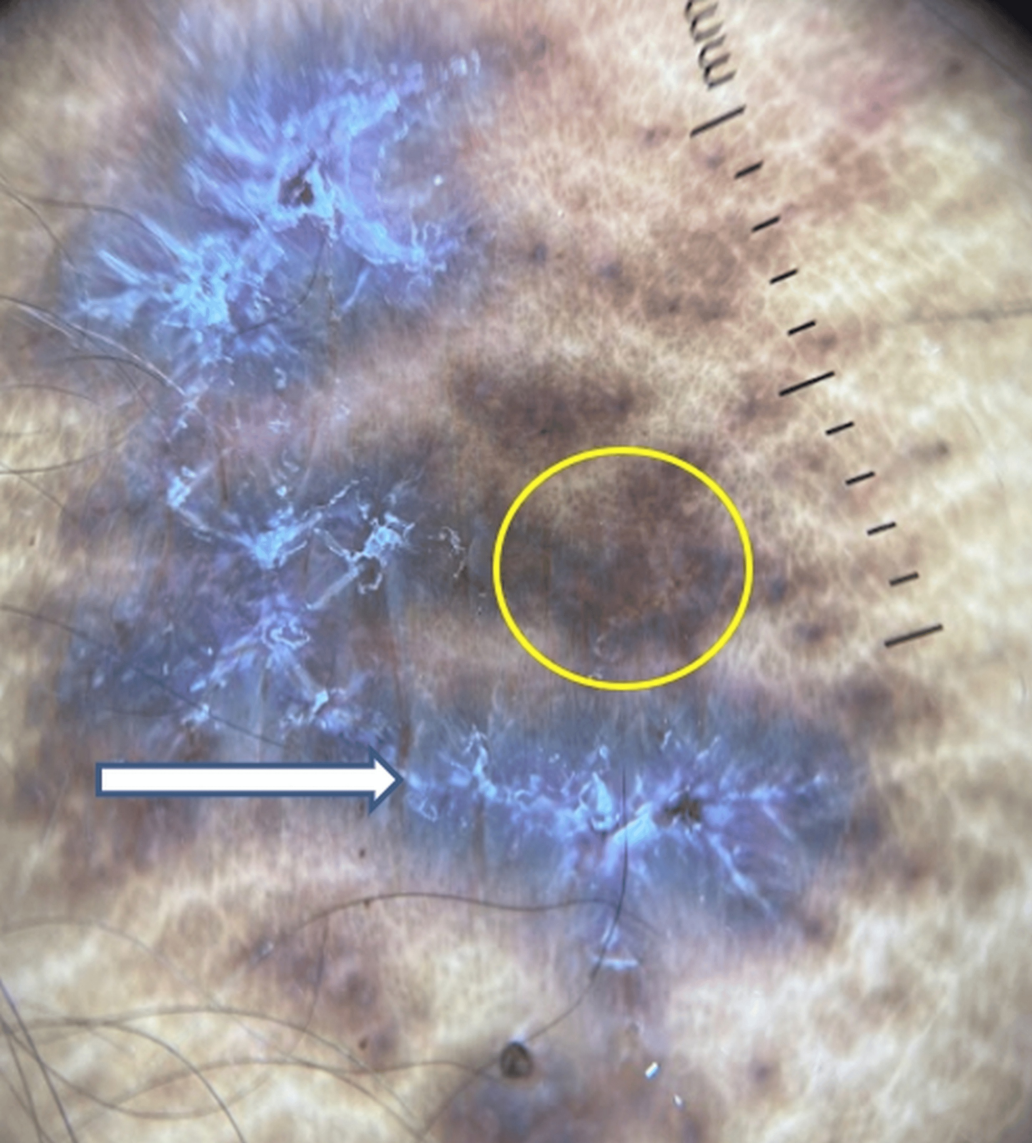
Dermoscopy showing lacy reticular streaks (arrow), with a diffuse brown pigmentation (circle) (DermLite, 10x) DermLite: DermLite, San Juan Capistrano, California, USA

Dermoscopy of the cutaneous lesions showed white, lacy reticular streaks in a radial and leaf venation pattern suggestive of Wickham striae, with diffuse brown pigmentation in the background (Figure 6).

Physical examination of other systems was unremarkable. The differential diagnosis of inverse erosive lichen planus, scrofuloderma, condyloma lata (cutaneous manifestation of secondary syphilis), pyoderma gangrenosum, hidradenitis suppurativa and cutaneous Crohn's disease was done.

**Figure 7:**
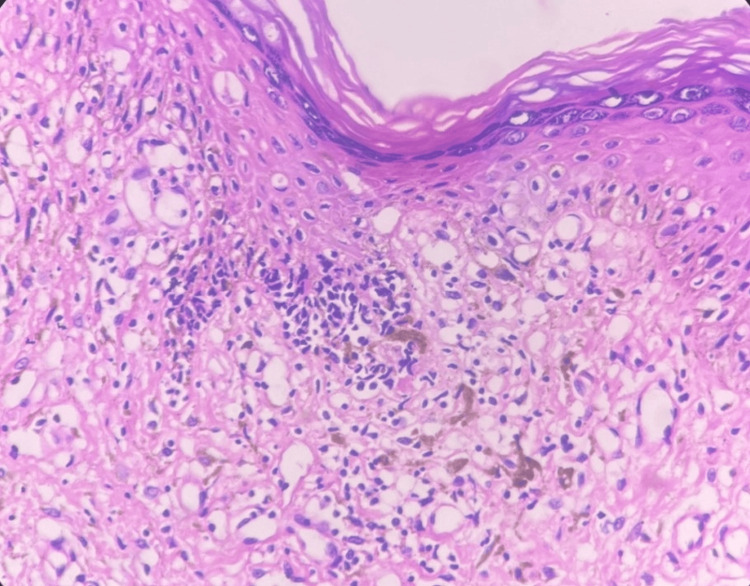
Histology showing focal interface dermatitis with melanin incontinence, along with a lymphohistiocytic infiltrate in the upper and mid dermis (H&E, x10) H&E: hematoxylin and eosin stain

Routine hematological and biochemical tests were normal, and serology for human immunodeficiency virus (HIV), hepatitis B, hepatitis C, and syphilis was negative. Mantoux test and a skin biopsy for CBNAAT (Cartridge-Based Nucleic Acid Amplification Test) to detect Mycobacterium tuberculosis were also negative. The biopsy from the ulcerated lesion on the groin and axilla sent for histopathology revealed focal interface dermatitis with melanin incontinence in the papillary dermis, along with a lymphohistiocytic infiltrate in the upper and mid dermis (Figure 7). The epidermis showed hyperkeratosis, acanthosis, and hypergranulosis. Acid fast bacillus stain was negative. 

**Figure 8:**
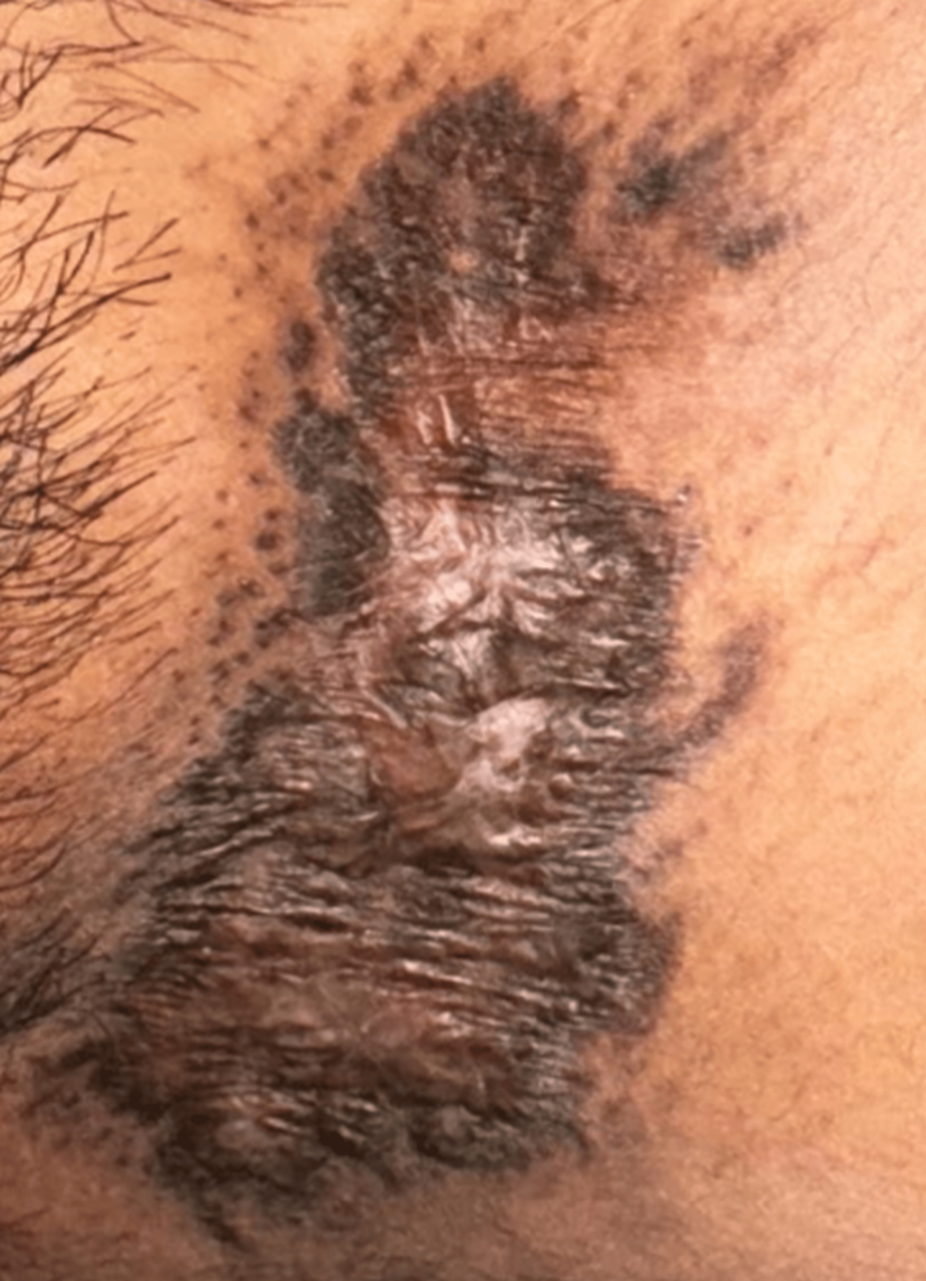
Inguinal lesion healing with hyperpigmentation and scarring at the eight-week follow-up.

**Figure 9:**
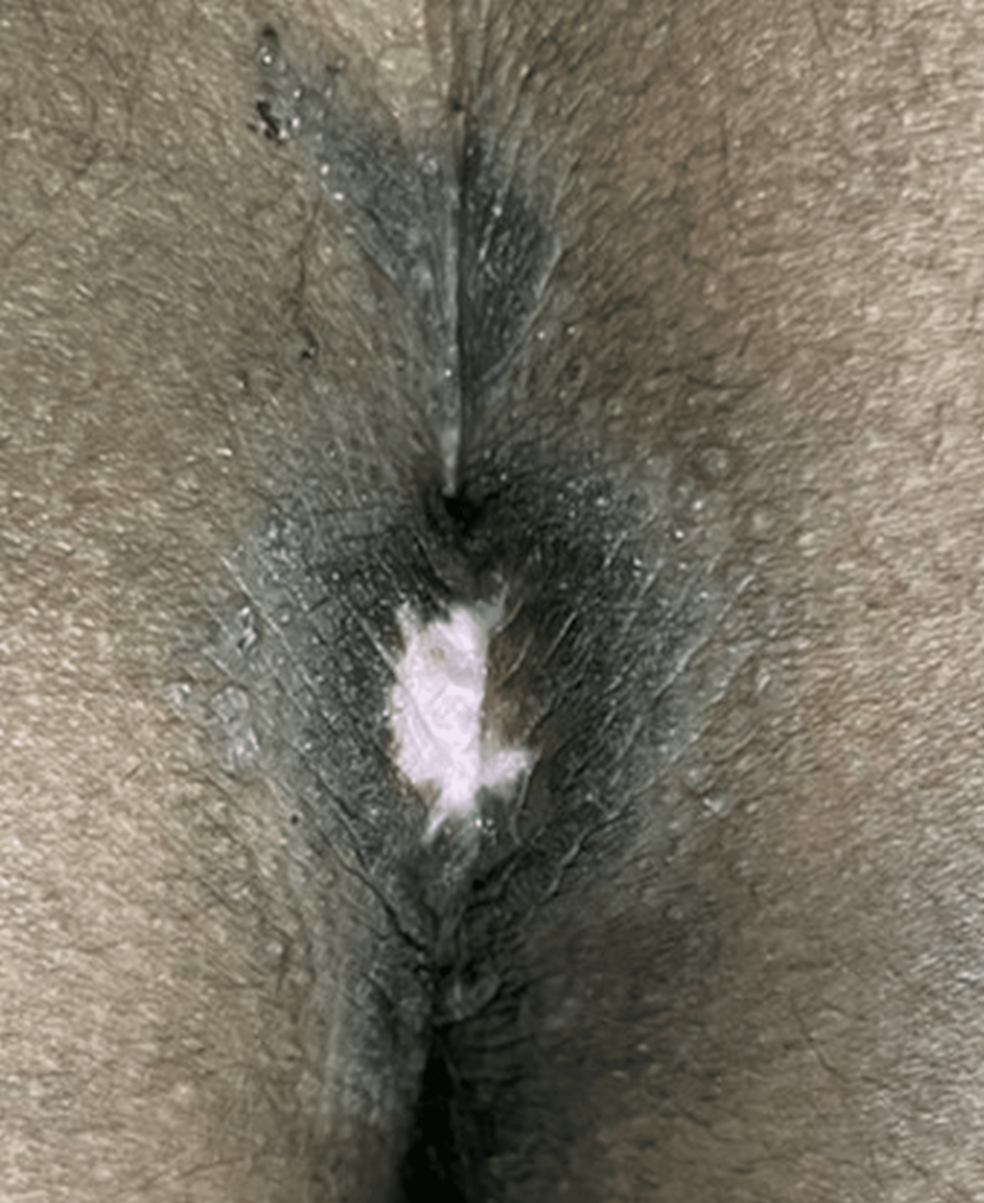
Perianal lesion showing pigmentation and scarring at the eight-week follow-up.

**Figure 10:**
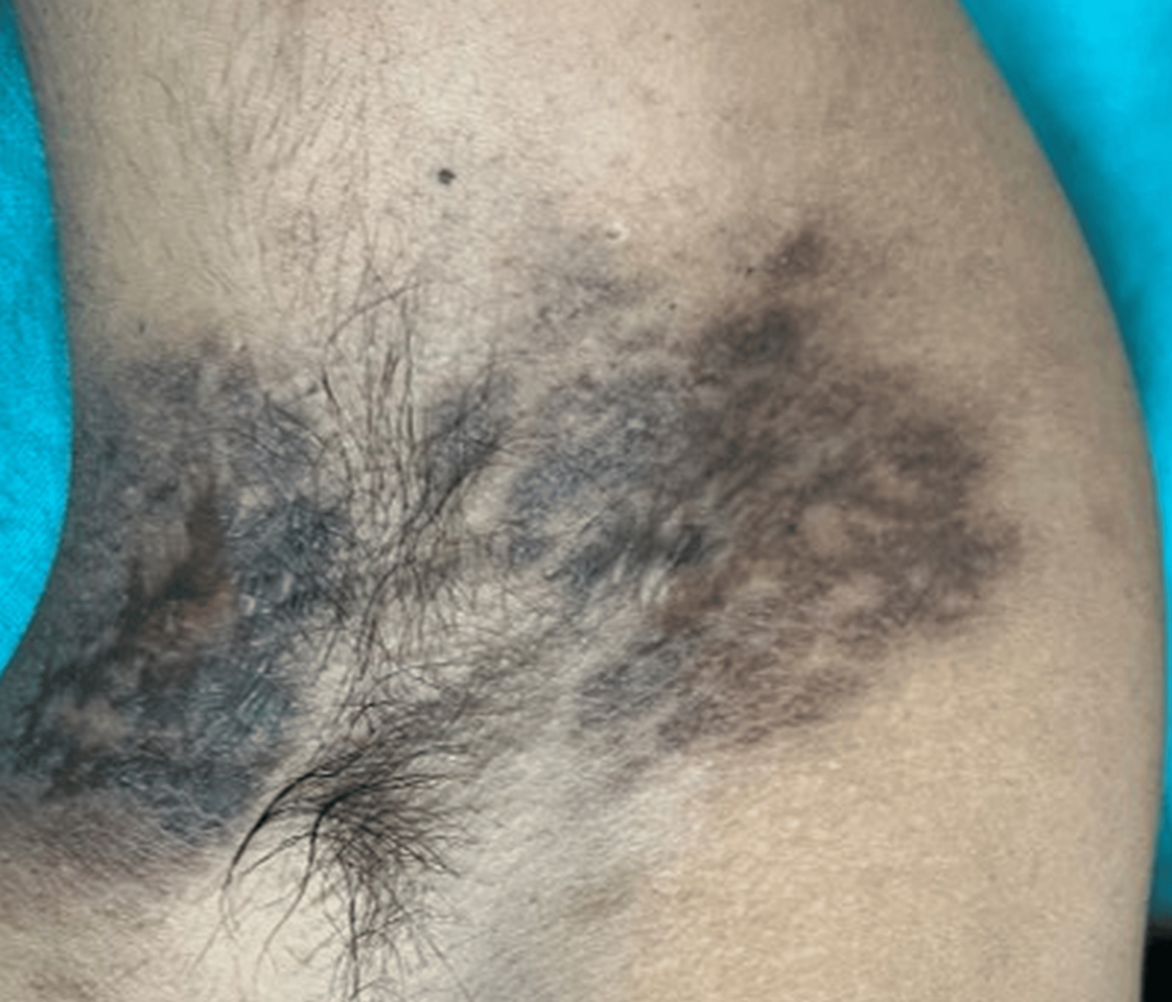
Axillary lesions showing hyperpigmentation at the eight-week follow-up.

Based on clinical, dermoscopic, and histopathological findings, a final diagnosis of inverse erosive lichen planus was made. The patient was initially treated with a short course of oral corticosteroids, specifically oral prednisolone at a dose of 0.75 mg/kg, tapered over four weeks. This was combined with topical clobetasol propionate cream 0.05% and oral antihistamines. Following treatment, complete resolution of the ulceration and associated symptoms was observed, with lesions healing with hyperpigmentation and mild scarring (Figures 8-10).

The patient has been maintained on isotretinoin, 20 mg and topical tacrolimus 0.1% with no recurrence for the past four months.

## Discussion

Lichen planus is a papulosquamous disorder that affects 0.5-1% of the population, most frequently occurring in middle-aged individuals [4]. It is considered rare in childhood, accounting for only 2%-3% of all lichen planus cases [7]. The disorder presents in various clinical forms, including plaque type, hypertrophic, atrophic, guttate, annular, linear, vesiculobullous, follicular, and erosive types, among others [8]. It can involve the skin, mucosa, or appendages, with rare instances of concurrent involvement across multiple anatomical sites, such as the oral cavity, scalp, nails, vulva, and vagina [9].

Although lichen planus can affect any part of the body, it rarely appears in areas such as the axillae, groins, and inframammary regions, as observed in our patient. Erosive lichen planus typically affects the oral mucosa and is the second most common variant of oral lichen planus, following the reticular variant. It is rare for the erosive variant to involve the skin, and when it does, it most commonly affects acral sites [3]. The involvement of flexural areas in erosive cutaneous lichen planus is extremely rare, ours being the first in an adolescent.

This variant poses a diagnostic challenge due to its resemblance to infectious and inflammatory dermatoses. Dermoscopy plays a critical role in the early and non-invasive diagnosis of lichen planus, aiding in the differentiation from other erosive dermatoses. The hallmark dermoscopic feature of lichen planus is the presence of Wickham striae, white, lacy, reticular streaks arranged in a radial or leaf venation pattern, often seen against a background of diffuse brown pigmentation [10]. This pattern is pathognomonic for lichen planus and provides an immediate diagnostic clue, even in atypical clinical presentations. In erosive lesions, dermoscopy can help identify subtle features masked by ulceration or erosion, facilitating timely diagnosis and early initiation of treatment.

Higgins et al. described a case similar to ours in a 57-year-old obese woman, who presented with red, sore erosive areas exclusively in flexural regions, with a violaceous hue and faint white streaks in the surrounding skin. The biopsy was suggestive of lichen planus, and the patient was managed with potent topical steroids [5]. Another case was reported by Eisman et al. involving a 79-year-old woman diagnosed with flexural lichen planus that was resistant to many forms of treatment. She later responded to a combination of oral thalidomide and topical tacrolimus 0.1% with complete healing of eroded and ulcerated areas in three months [4]. The third case was that of a 58-year-old male farmer with intensely itchy dermatitis over the inguinal region, with the lesion showing eroded hypertrophic, lilac-colored tissue. This patient was treated with cyclosporin A at 3 mg/kg/day, resulting in rapid improvement and complete symptom remission within two months. However, recurrence was noted upon discontinuation of treatment [6].

Reported treatment options for erosive lichen planus include topical corticosteroids, tetracyclines, retinoids, cyclosporin, griseofulvin, dapsone, azathioprine, and extracorporeal photochemotherapy [11]. This variant of lichen planus is notoriously difficult to treat and often follows a chronic course with a low likelihood of spontaneous resolution.

## Conclusions

This case highlights the importance of considering inverse erosive lichen planus in the differential diagnosis of erosive dermatoses in flexural regions. It underscores the importance of dermoscopy as an essential tool in the diagnostic armamentarium for erosive dermatoses, particularly in challenging intertriginous presentations. Timely diagnosis, guided by histopathological and dermoscopic findings, enables the early initiation of treatment. Our case demonstrates that a combination of corticosteroids, topical immunomodulators, and retinoids can lead to sustained remission in cases of inverse erosive lichen planus. Further reports are essential to understand the pathogenesis, clinical spectrum, and optimal treatment strategies for this rare variant of lichen planus.
